# Role of renal function in risk assessment of target non-attainment after standard dosing of meropenem in critically ill patients: a prospective observational study

**DOI:** 10.1186/s13054-017-1829-4

**Published:** 2017-10-21

**Authors:** Lisa Ehmann, Michael Zoller, Iris K. Minichmayr, Christina Scharf, Barbara Maier, Maximilian V. Schmitt, Niklas Hartung, Wilhelm Huisinga, Michael Vogeser, Lorenz Frey, Johannes Zander, Charlotte Kloft

**Affiliations:** 1 Department of Clinical Pharmacy and Biochemistry, Institute of Pharmacy, Freie Universitaet Berlin, Kelchstrasse 31, 12169 Berlin, Germany; 2 Graduate Research Training Program PharMetrX, Berlin/Potsdam, Germany; 3 Department of Anaesthesiology, University Hospital, LMU Munich, Munich, Germany; 4 Institute of Laboratory Medicine, University Hospital, LMU Munich, Munich, Germany; 5 Institute of Pharmacy and Molecular Biotechnology, University of Heidelberg, Heidelberg, Germany; 6 Institute of Mathematics, Universitaet Potsdam, Potsdam, Germany

**Keywords:** β-Lactam, Intensive care, Pharmacokinetics/Pharmacodynamics, Target attainment, Renal function, Risk assessment tool, Continuous renal replacement therapy

## Abstract

**Background:**

Severe bacterial infections remain a major challenge in intensive care units because of their high prevalence and mortality. Adequate antibiotic exposure has been associated with clinical success in critically ill patients. The objective of this study was to investigate the target attainment of standard meropenem dosing in a heterogeneous critically ill population, to quantify the impact of the full renal function spectrum on meropenem exposure and target attainment, and ultimately to translate the findings into a tool for practical application.

**Methods:**

A prospective observational single-centre study was performed with critically ill patients with severe infections receiving standard dosing of meropenem. Serial blood samples were drawn over 4 study days to determine meropenem serum concentrations. Renal function was assessed by creatinine clearance according to the Cockcroft and Gault equation (CLCR_CG_). Variability in meropenem serum concentrations was quantified at the middle and end of each monitored dosing interval. The attainment of two pharmacokinetic/pharmacodynamic targets (100%T_>MIC_, 50%T_>4×MIC_) was evaluated for minimum inhibitory concentration (MIC) values of 2 mg/L and 8 mg/L and standard meropenem dosing (1000 mg, 30-minute infusion, every 8 h). Furthermore, we assessed the impact of CLCR_CG_ on meropenem concentrations and target attainment and developed a tool for risk assessment of target non-attainment.

**Results:**

Large inter- and intra-patient variability in meropenem concentrations was observed in the critically ill population (*n* = 48). Attainment of the target 100%T_>MIC_ was merely 48.4% and 20.6%, given MIC values of 2 mg/L and 8 mg/L, respectively, and similar for the target 50%T_>4×MIC_. A hyperbolic relationship between CLCR_CG_ (25–255 ml/minute) and meropenem serum concentrations at the end of the dosing interval (C_8h_) was derived. For infections with pathogens of MIC 2 mg/L, mild renal impairment up to augmented renal function was identified as a risk factor for target non-attainment (for MIC 8 mg/L, additionally, moderate renal impairment).

**Conclusions:**

The investigated standard meropenem dosing regimen appeared to result in insufficient meropenem exposure in a considerable fraction of critically ill patients. An easy- and free-to-use tool (the MeroRisk Calculator) for assessing the risk of target non-attainment for a given renal function and MIC value was developed.

**Trial registration:**

Clinicaltrials.gov, NCT01793012. Registered on 24 January 2013.

**Electronic supplementary material:**

The online version of this article (doi:10.1186/s13054-017-1829-4) contains supplementary material, which is available to authorized users.

## Background

Severe infections remain a major issue in the intensive care unit (ICU) because of their high prevalence and high mortality rates among critically ill patients [1]. Hence, rational antibiotic therapy is especially important in this vulnerable population. Apart from an appropriate activity spectrum and early initiation of antibiotic therapy, a dosing regimen leading to adequate therapeutic antibiotic concentrations and exposure is crucial [2–5]. Adequate antibiotic exposure not only has been found to improve clinical success but also has been suggested to reduce resistance development [6, 7]. At the same time, pathophysiological changes in critically ill patients, including organ dysfunction or altered fluid balance, might substantially influence antibiotic concentrations and increase the risk of inadequate antibiotic exposure. As a second challenge, infections in these patients are often caused by pathogens with lower susceptibility (i.e., higher minimum inhibitory concentration [MIC]) than in other clinical settings [8–11].

Meropenem is a broad-spectrum carbapenem β-lactam antibiotic frequently used to treat severe bacterial infections in critically ill patients, such as those with severe pneumonia, complicated intra-abdominal infections, complicated skin and soft tissue infections, or sepsis [12]. For these indications, the approved standard dosing regimens for adults (intact renal function [RF]) include 500 mg or 1000 mg administered as short-term infusions every 8 h; for other indications, doses up to 2000 mg are recommended [12]. Meropenem is a hydrophilic molecule with very low plasma protein binding of approximately 2% [13]. It is excreted primarily via the kidney, predominantly by glomerular filtration but also by active tubular secretion [14]. Meropenem has been shown to be readily dialysable and effectively removed by haemodialysis [15–17]. As a β-lactam antibiotic, meropenem shows time-dependent activity; that is, its antibacterial activity is linked to the percentage of time that meropenem concentrations exceed the MIC value of a pathogen (%T_>MIC_) [18]. The attainment of the pharmacokinetic/pharmacodynamic (PK/PD) index %T_>MIC_ has been associated with clinical success in patients treated with meropenem [19–21]. For example, Ariano et al. demonstrated that the probability of clinical response was 80% when %T_>MIC_ was 76–100 in febrile neutropenic patients with bacteraemia but only 36% when %T_>MIC_ was between 0 and 50 [20].

Previous studies have revealed large inter-patient variability in meropenem concentrations after standard dosing in critically ill patients [22–24], which resulted in inadequate meropenem exposure in a relevant fraction of patients [23, 25]. However, in most of these studies, only limited numbers of patients and/or rather homogeneous patient sub-groups have been investigated. Hence, the identified variability in meropenem exposure might not have adequately reflected a typically heterogeneous critically ill population. In previous analyses, RF has been shown to be a major cause of variability in meropenem exposure [23, 24, 26–31] and, as a consequence, to be influential on the attainment of specific target concentrations [25, 32, 33]. However, the impact of kidney function on target attainment has been assessed primarily for distinct RF classes but not yet in a coherent quantitative framework for a population covering the full spectrum of RF ranging from dialysis/severe renal impairment (RI) to augmented renal clearance.

The aims of this study were (1) to quantify inter- and intra-individual variability of meropenem serum concentrations in a heterogeneous critically ill population covering the full spectrum of RF classes after meropenem standard dosing, (2) to investigate the attainment of two different PK/PD targets, (3) to assess the impact of RF on meropenem exposure and consequently target attainment and (4) ultimately to develop an easy-to-use risk assessment tool allowing identification and quantification of the risk of target non-attainment for a particular patient on the basis of the patient’s RF.

## Methods

### Clinical study

This prospective observational study was conducted at three ICUs within the Department of Anaesthesiology, University Hospital, LMU Munich, Germany. The study protocol (ClinicalTrials.gov identifier NCT01793012) was approved by the Institutional Review Board of the Medical Faculty of the LMU Munich, Germany. Criteria for inclusion comprised the presence of severe infection (confirmed or suspected by clinical assessment), age ≥ 18 years and therapy with meropenem (including possible de-escalation; clinical assessment independent from the study). Patients were excluded in case of a planned hospitalisation < 4 days or meropenem administration > 48 h prior to study start. Written informed consent to participate was obtained from all patients or their legal representatives. All patients received standard doses of meropenem as 30-minute infusions three times per day (*see* Additional file 1: Study design, Figure S1a). Multiple arterial blood samples were collected for the quantification of meropenem concentrations over a study period of 4 days. Intensive sample collection was performed during all three dosing intervals of study day 1 and during the first dosing interval of study days 2–4. An additional single minimum meropenem concentration (C_min_) sample before the next dose was collected for the third dosing interval of days 2 and 3. The planned sampling time points per intensively monitored dosing interval were as follows: 15 minutes, 30 minutes, 1.5 h, 4 h, and 8 h (directly before next dose; C_min_) after the start of infusion (*see* Additional file 1: Study design, Figure S1b). The exact sampling time points were recorded by the medical staff. In addition, patient-specific data such as diagnosis, demographics, disease scores and laboratory data (e.g., serum creatinine) were recorded during the study period. Creatinine clearance was estimated according to the Cockcroft and Gault equation (CLCR_CG_ [34]) on the basis of daily measured serum creatinine (Jaffe assay):

$$ \mathrm{CLC}{\mathrm{R}}_{\mathrm{CG}}\left[\frac{\mathrm{ml}}{\min}\right]=\frac{\left(140-\mathrm{age}\ \left[\mathrm{years}\right]\right)\cdot \mathrm{body}\  \mathrm{weight}\left[\mathrm{kg}\right]}{72\cdot \mathrm{serum}\  \mathrm{creatinine}\left[\frac{\mathrm{mg}}{\mathrm{dl}}\right]}\cdot \left(0.85\ \mathrm{if}\  \mathrm{female}\right) $$

In addition, pathogens identified in specimens collected from the patients (between 3 days before and 3 days after the study period) were recorded.

### Bioanalytical method for meropenem concentration

Blood samples were immediately sent to the Institute of Laboratory Medicine, University Hospital, LMU Munich and centrifuged. Serum samples were stored at −80 °C until total meropenem serum concentration was quantified by using a validated liquid chromatography-tandem mass spectrometry method described previously [35]. Briefly, sixfold deuterated meropenem was used as an internal standard, and validation revealed good analytical performance, with an inaccuracy of less than or equal to ± 4% relative error and imprecision ≤ 6% coefficient of variation (CV).

### Variability of meropenem concentrations

To quantify inter- and intra-individual variability of meropenem serum concentrations, measured C_min_ values were first analysed without regard to the actual heterogeneous sampling time points or administered doses. Inter-individual variability was evaluated by a summary statistical analysis of all available C_min_ values; for description of intra-individual variability, the ratios of the maximum and minimum C_min_ values $\left(\frac{{\mathrm{C}}_{\min \_\max }}{{\mathrm{C}}_{\min \_\min }}\right)$ of all dosing intervals monitored within a patient were statistically summarised. Summary statistics included median, range, 95% CI and %CV.

In order to exclude a potential impact of dose- and sampling time point-related variability on the meropenem minimum concentrations, dose-normalised meropenem concentrations (to a dose of 1000 mg, assuming linear PK) at two specific time points (4 h [C_4h_] and 8 h [C_8h_] after infusion start) were calculated, and the variability was evaluated as described above. C_4h_ and C_8h_ values were determined by linear regression (if more than two data points) or linear interpolation (if two data points) of the logarithmised data in the declining phase of each concentration-time profile. In case of a coefficient of determination (*R* ^2^) < 0.9, being associated with two distinct phases in the declining part of the concentration-time profile, a separate linear interpolation/regression was performed for each of these phases.

### Pharmacokinetic/pharmacodynamic target attainment

To evaluate the achievement of therapeutically adequate meropenem serum concentrations, PK/PD target attainment was assessed for a broad MIC range from 0.25 mg/L to 8 mg/L, with a special focus on MIC 2 mg/L and MIC 8 mg/. The two values are common European Committee on Antimicrobial Susceptibility Testing (EUCAST) susceptible/intermediate (S/I) and intermediate/resistant (I/R) MIC breakpoints for relevant bacteria, such as Enterobacteriaceae, *Pseudomonas* spp. or *Acinetobacter* spp. [36]. The target 100%T_>MIC_ (i.e., meropenem serum concentrations exceeding one times the MIC for the entire dosing interval) was selected because it has previously been shown to improve clinical cure and bacteriological eradication in patients with serious bacterial infections treated with β-lactam antibiotics [20, 37]. In accordance with other studies, 50%T_>4×MIC_ (i.e., meropenem serum concentration exceeding four times the MIC for half of the dosing interval) was chosen as a second target [38–40]. Owing to the negligible protein binding of meropenem (2%), total meropenem serum concentrations were used for all analyses [13, 41].

To evaluate the attainment of the targets 100%T_>MIC_ and 50%T_>4×MIC_, the predicted C_4h_ and C_8h_ values of each dosing interval were evaluated regarding the achievement of the above-mentioned thresholds (one or four times the MIC breakpoints) for all patients not undergoing continuous renal replacement therapy (CRRT). Additionally, target attainment was evaluated for a dose of 2000 mg meropenem based on the extrapolated C_4h_ and C_8h_ values (assuming linear PK). Dosing was considered adequate if the target was attained in ≥ 90% of the monitored dosing intervals [41].

### Impact of renal function on meropenem exposure and target attainment

To investigate the impact of RF on meropenem exposure, CLCR_CG_ was related to C_4h_ and C_8h_ values (at patient level using the median individual CLCR_CG_ of a patient, and at sample level using single CLCR_CG_ values). For non-CRRT patients, the relationship between CLCR_CG_ and C_8h_ values was quantified by weighted linear least squares regression in double logarithmic scale $\left({\mathrm{C}}_{8\mathrm{h}}=\upalpha \cdot \frac{1}{{\left(\mathrm{CLC}{\mathrm{R}}_{\mathrm{C}\mathrm{G}}\right)}^{\upbeta}}\right)$. For further details, *see* Additional file 2: Regression model for risk calculation.

Target attainment at sample level was stratified by the following classes of RF or RI on the basis of CLCR_CG_ [42–44]: severe RI 15–29 ml/minute, moderate RI 30–59 ml/minute, mild RI 60–89 ml/minute, normal RF 90–129 ml/minute and augmented RF ≥ 130 ml/minute. All analyses described here and previously were performed using the software R, version 3.3.2 (R Foundation for Statistical Computing, Vienna, Austria).

### Risk assessment tool

A tool for the risk assessment of target non-attainment based on the RF was developed using Excel 2016 software with Visual Basic for Applications (Microsoft Corporation, Redmond, WA, USA). In the Excel tool, the quantified CLCR_CG_-C_8h_ relationship for non-CRRT patients, the prediction interval around this relationship and the computation of the risk of target (100%T_>MIC_) non-attainment for given CLCR_CG_ and MIC values were implemented. For further details, *see* Additional file 2: Regression model for risk calculation.

## Results

### Clinical study

#### Patient characteristics

A total of 48 patients (27 male, 21 female) were included in the study (*see* Table 1). Of these patients, 83% suffered from sepsis, which was most frequently caused by pneumonia or peritonitis (75% or 20% of the sepsis patients, respectively). Pathogens detected in the patients comprised Enterobacteriaceae, non-fermenters (e.g., *Pseudomonas* spp.), *Staphylococcus* spp*.*, *Streptococcus* spp*.*, *Enterococcus* spp., *Bacillus* spp., *Clostridium* spp*.*, *Bacteroides* spp., *Mycoplasma* spp*.*, *Candida* spp. and *Aspergillus* spp*.* The patient group covered broad ranges of age (24–84 years), body mass index (16–49 kg/m^2^) and severity of illness (Acute Physiology and Chronic Health Evaluation II [APACHE II] score 11–42). RF determined by CLCR_CG_ was highly variable, ranging from severely impaired to augmented RF (first study day 24.8–191 ml/minute). Seven patients received CRRT, and six patients underwent extracorporeal membrane oxygenation (ECMO). Twenty-eight patients were post-lung or post-liver transplant recipients.

**Table 1:**
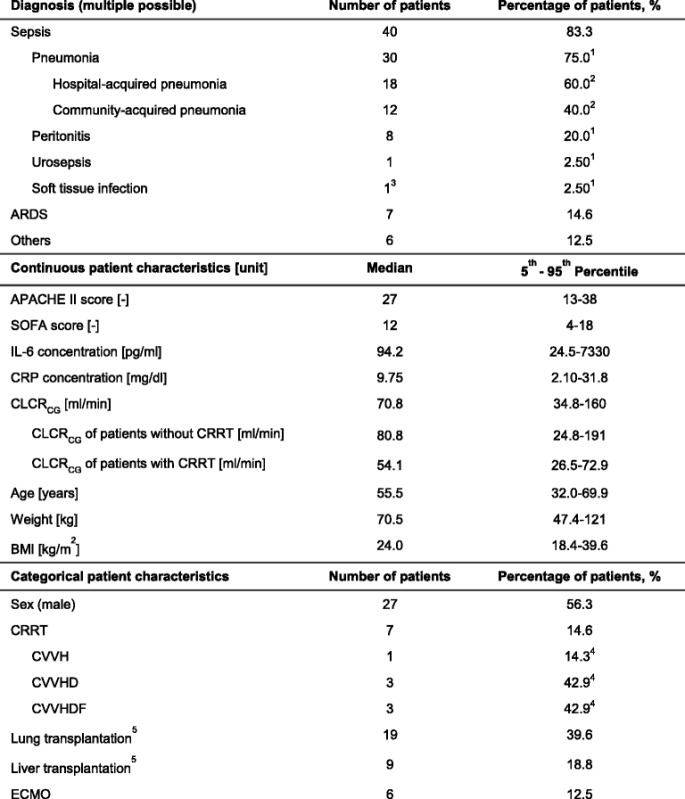
Patient characteristics on study day 1

*Abbreviations*: *APACHE II* Acute Physiology And Chronic Health Evaluation II [53], *ARDS* Acute respiratory distress syndrome, *BMI* Body mass index, *CLCR*_*CG*_ Creatinine clearance estimated according to Cockcroft and Gault equation [34], *CRP* C-reactive protein, *CRRT* Continuous renal replacement therapy, *CVVH* Continuous venovenous haemofiltration, *CVVHD* Continuous venovenous haemodialysis, *CVVHDF* Continuous venovenous haemodiafiltration, *ECMO* Extracorporeal membrane oxygenation, *IL-6* Interleukin 6, *SOFA* Sepsis-related Organ Failure Assessment [54]

^1^In relation to total number of patients with sepsis

^2^In relation to total number of patients with pneumonia

^3^Abdominal wall abscess

^4^In relation to total number of patients with CRRT

^5^Transplant within last 28 days

#### Meropenem dosing and sampling

**Fig. 1:**
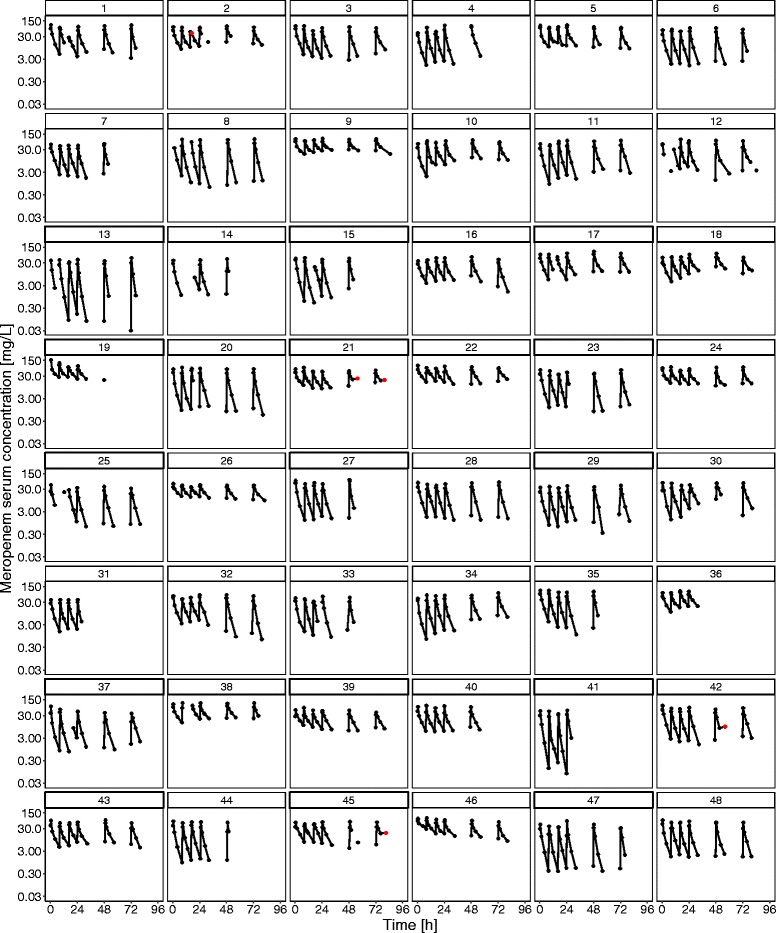
Individual meropenem serum concentration-time profiles. *Number above individual plot* is patient identifier. *Circles* represent measured meropenem concentrations. *Red circles* represent meropenem concentrations excluded from analyses (0.36%; *see text*). *Lines* represent connection of consecutively sampled meropenem concentrations; that is, gaps represent non-monitored dosing intervals or missing planned meropenem concentration measurements

During the study period, patients were treated with 1000 mg (*n*_patients_ = 47) or 2000 mg (*n*_patients_ = 1) of meropenem administered as 30-minute infusions approximately every 8 h (median 8 h, 95% CI 6.94–9.19 h). A total of 1376 blood samples (median per patient 31) were taken during 349 dosing intervals (median per patient 8, range per patient 4–8). Of the measurements, 23.5% (*n* = 324) were C_min_ samples, which were collected 7.92 h (median) after infusion start (95% CI 6.85–9.08 h). Very few serum concentrations (0.36% of data) revealed an implausible increase in the terminal part of the concentration-time profiles and were therefore excluded from the data analyses (*red* data points in Fig. 1).

### Variability of meropenem concentrations

**Fig. 2:**
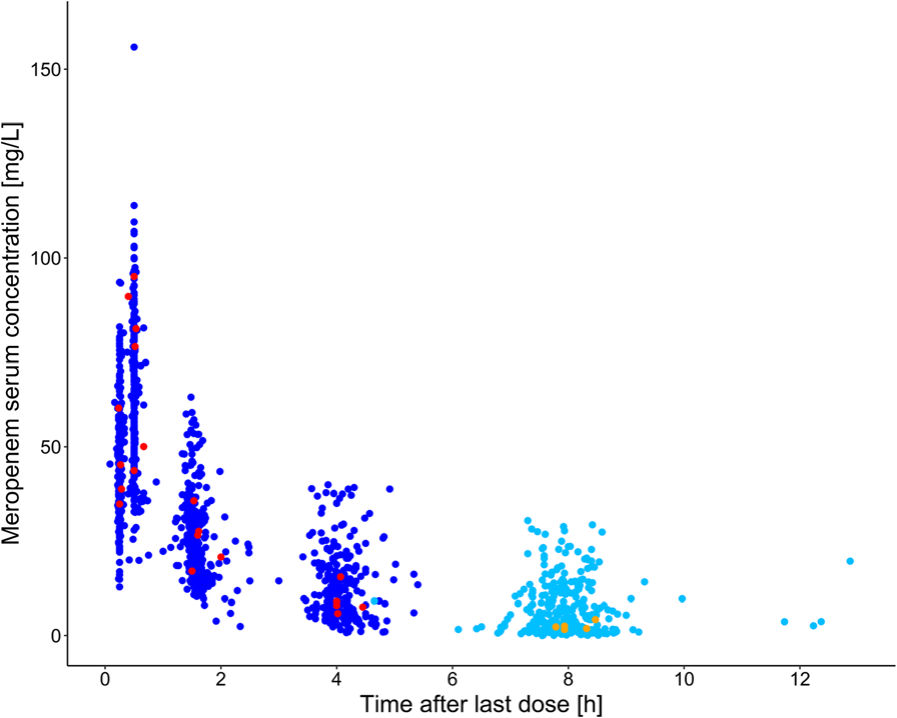
Meropenem serum concentrations vs. time after last dose (*n* = 48 patients). *Dark blue/red circles* represent concentrations of patients treated with 1000 mg/2000 mg meropenem. *Light blue/orange circles* represent measured meropenem serum concentration values at the end of the actual dosing interval among patients treated with 1000 mg/2000 mg meropenem

Large inter-individual variability was observed for both the observed C_min_ values (*see* Fig. 2) and the calculated concentrations C_8h_ and C_4h_ (*see* Table 2). Whereas inter-individual variability in C_min_ and C_8h_ was particularly large, varying in both concentrations by up to a factor of approximately 1000 between patients, C_4h_ values were slightly less variable (C_min_ range 0.03–30.0 mg/L, 104 CV%; C_8h_ range 0.0426–30.0 mg/L, 110 CV%; C_4h_ range 0.933–43.3 mg/L, 69.9 CV%). Apart from inter-individual variability, large intra-individual variability was identified (*see* Table 2). Particularly C_min_ (*see* Fig. 1) and C_8h_ values showed large variability, with concentrations varying in median by twofold to more than tenfold within a patient (range of ratios $\frac{{\mathrm{C}}_{\min \_\max }}{{\mathrm{C}}_{\min \_\min }}$: 1.3–10.9, range of ratios $\frac{{\mathrm{C}}_{8\mathrm{h}\_\max }}{{\mathrm{C}}_{8\mathrm{h}\_\min }}$: 1.22–11.4). Intra-individual variability in C_4h_ values was slightly lower, but the C_4h_ values within a patient still varied up to more than fivefold (range of ratios $\frac{{\mathrm{C}}_{4\mathrm{h}\_\max }}{{\mathrm{C}}_{4\mathrm{h}\_\min }}$: 1.10–5.47).

**Table 2:**
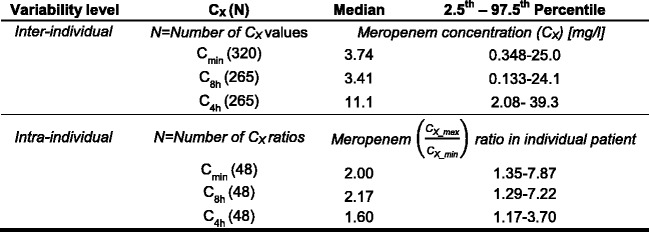
Inter- and intra-individual variability of meropenem concentrations at specific time points

*Abbreviations*: *C*_*min*_ Measured meropenem serum concentration at end of actual dosing interval, *C*_*X*_ Meropenem serum concentration at specific time point X of concentration-time profile

### Pharmacokinetic/pharmacodynamic target attainment

For infections in non-CRRT patients with pathogens of MIC 2 mg/L, both investigated targets were attained in approximately half of the dosing intervals monitored, with slightly higher attainment for the 50%T_>4×MIC_ target (56%) than for the 100%T_>MIC_ target (48%; *see* Table 3). When extrapolating the data to a dose of 2000 mg, target attainment was substantially higher, with 91% and 78% for the targets 50%T_>4×MIC_ and 100%T_>MIC_, respectively (*see* Additional file 3: PK/PD target attainment, Table S2).

**Table 3:**
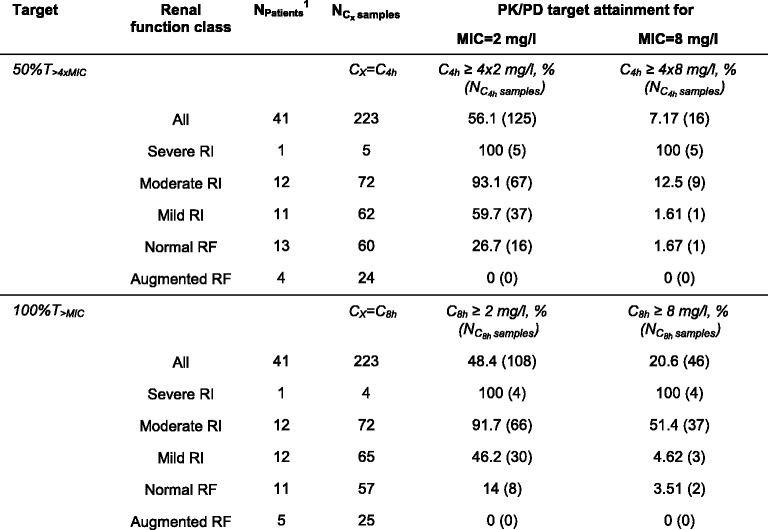
Pharmacokinetic/pharmacodynamic target attainment for all patients not receiving continuous renal replacement therapy and stratified by renal function

*Abbreviations*: *CLCR*_*CG*_ Creatinine clearance estimated according to Cockcroft and Gault equation [34], *CRRT* Continuous renal replacement therapy, *C*_*X*_ Concentration at specific time point X of concentration-time profile, *I/R* Intermediate/resistant, *PK/PD* Pharmacokinetic/pharmacodynamic, *RF* Renal function, *RI* Renal impairment, *S/I* Susceptible/intermediate

^1^Patients were assigned to a renal function class on the basis of their median individual CLCR_CG_ at the time of C_4h_ or C_8h_ determination

Given an MIC of 8 mg/L, the target 100%T_>MIC_ was attained only in about one-fifth of the monitored meropenem dosing intervals; attainment of the target 50%T_>4×MIC_ was very low (7%; *see* Table 3). When extrapolating to a dose of 2000 mg, the attainment of 100%T_>MIC_ was approximately twice as high as for a dose of 1000 mg (38.1% vs. 20.6%); the attainment of 50%T_>4×MIC_ was even about four times as high (27.4% vs. 7.17%) (*see* Additional file 3: PK/PD target attainment, Table S2). For doses of 1000 mg and 2000 mg, target attainment for the full MIC range from 0.25 mg/L to 8 mg/L is summarised in Additional file 3: PK/PD target attainment.

### Impact of renal function on meropenem exposure and target attainment

**Fig. 3:**
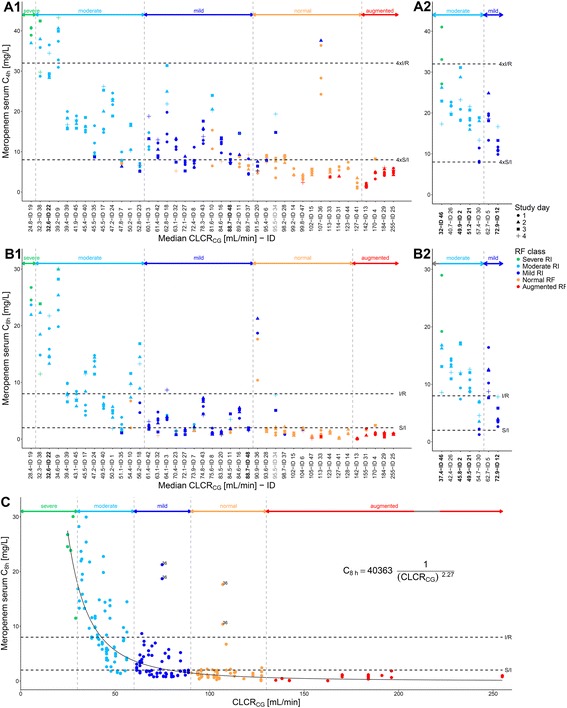
Relationship between meropenem serum concentration and creatinine clearance. Meropenem serum concentrations 4 h (C_4h_) (**a1**, **a2**) and 8 h (C_8h_) (**b1**, **b2**, **c**) after start of infusion in non-CRRT (**a1**, **b1**, **c**) and CRRT (**a2**, **b2**) patients vs. median individual CLCR_CG_ (patient level; **a**, **b**) or vs. all single CLCR_CG_ (sample level; **c**) of the patients. *Tick mark of x-axis* (**a**, **b**) represents median individual CLCR_CG_ at time of determined C_4h_ or C_8h_ value. *Bold tick mark labels* (**a**, **b**) represent ECMO patients. *Grey tick mark labels* (**a1**, **b1**) represent patient example mentioned in “Impact of renal function on meropenem exposure and target attainment” section of main text. *Coloured symbols* (**a-c**) represent renal function class of a patient at time of determined C_4h_ or C_8h_ value. *Shaped symbols* (**a**, **b**) represent study day on which C_4h_ or C_8h_ value was determined. *Dashed vertical lines/horizontal arrows* (**a-c**) represent separation of renal function classes. *Dashed horizontal lines* (**a-c**) represent EUCAST MIC breakpoints for Enterobacteriaceae, *Pseudomonas* spp. or *Acinetobacter* spp. (S/I 2 mg/L, I/R 8 mg/L [36]). *Data points labelled with 36* (**c**) represent four C_8h_ values of patient 36. *Black curve* (**c**) represents quantified hyperbolic relationship between CLCR_CG_ and C_8h_ values, excluding data of patient 36. *Abbreviations: CLCR*_*CG*_ Creatinine clearance estimated according to Cockcroft and Gault [34]; *CRRT* Continuous renal replacement therapy; *C*_*4h*_ Meropenem serum concentration at 4 h after infusion start; *C*_*8h*_ Meropenem serum concentration at 8 h after infusion start; *ECMO* Extracorporeal membrane oxygenation; *EUCAST* European Committee on Antimicrobial Susceptibility Testing; *ID* Patient identifier; *I/R* Intermediate/resistant; *MIC* Minimum inhibitory concentration; *S/I* Susceptible/intermediate

In addition to the large inter- and intra-patient variability in meropenem exposure (i.e., C_4h_ values [*see* Fig. 3a, *y*-axis] and C_8h_ values [*see* Fig. 3b, *y*-axis]), large variability was also observed for RF, with representatives in all RF classes from severe RI to augmented RF (*see* Fig. 3, *x*-axes). In addition to the 41 non-CRRT patients, 7 CRRT patients were investigated. Whereas RF was stable (i.e., constant RF class) within the monitored study period for half of the patients (*n* = 24), RF of the other half changed between two (*n*_patients_ = 21) or even three (*n*_patients_ = 3) classes of RF. Already at the patient level, a strong dependency between median individual CLCR_CG_ and C_4h_ (*see* Fig. 3a1) and C_8h_ (*see* Fig. 3b1) of the patients was found, interestingly also for the CRRT patients (*see* Fig. 3a2, b2). Also of note, in patients undergoing ECMO, meropenem concentrations were comparable with non-ECMO patients regarding their median individual CLCR_CG_. Moreover, within most of the individuals with changing RF, the same tendency of higher meropenem exposure for decreased RF was observed; for example, patient 34 had worsening of RF and at the same time increasing meropenem exposure across the 4 study days (*see grey tick mark label* in Fig. 3a1, b1). At the sample level (i.e., when relating all single CLCR_CG_ values as a continuous variable to meropenem exposure [C_8h_]), a distinct relation was found, which was described by the hyperbolic function ${\mathrm{C}}_{8\mathrm{h}}=40363\cdot \frac{1}{{\left(\mathrm{CLC}{\mathrm{R}}_{\mathrm{C}\mathrm{G}}\right)}^{2.27}}$ (*see* Fig. 3c; without C_8h_ values of patient 36). Four C_8h_ values of one patient (patient 36) were excluded from the regression because they were considerably larger than those of the remaining patients with similar RF; when including the four values of this patient, the predicted C_8h_ values in the investigated CLCR_CG_ range changed only negligibly for all metrics (quantified CLCR_CG_-meropenem exposure relationship, 95% CI, 95% prediction interval) (*see* Additional file 2: Regression model for risk calculation, Figure S2).

In non-CRRT patients, stratification of target attainment by the RF classes identified augmented RF to mild RI (CLCR_CG_ > 130–60 ml/minute) as a risk factor for non-attainment of both targets (target attainment 0–46.2% for 100%T_>MIC_, 0–59.7% for 50%T_>4×MIC_) (*see* Table 3) for infections with pathogens of MIC 2 mg/L. Given an MIC of 8 mg/L, meropenem treatment resulted in reliable target attainment only in the presence of severe RI (CLCR_CG_ 15–29 ml/minute); thus, already moderate RI (CLCR_CG_ 30–59 ml/minute) was identified as a risk factor for target non-attainment (target attainment for moderate RI 51.4% for 100%T_>MIC_, 12.5% for 50%T_>4×MIC_).

### Risk assessment tool

**Fig. 4:**
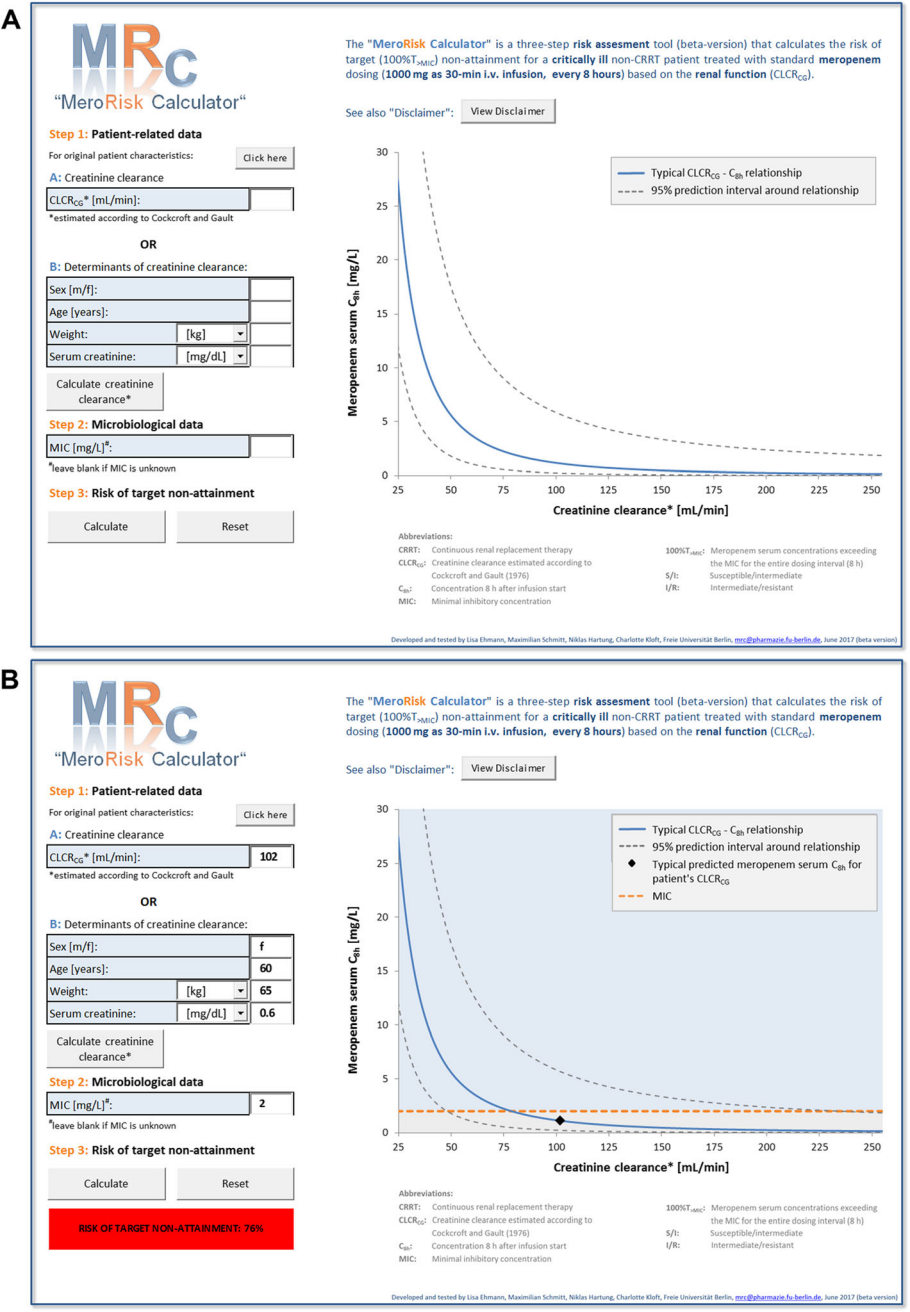
Graphical user interface of the MeroRisk Calculator. **a** Display when opening the tool (i.e., without any entries). **b** Display after risk calculation for a specific patient: female, aged 60 years, body weight 65 kg, serum creatinine 0.6 mg/dl, infected with pathogen of MIC 2 mg/L. *Abbreviations: CLCR*_*CG*_ Creatinine clearance estimated according to Cockcroft and Gault equation [34], *CRRT* Continuous renal replacement therapy, *C*_*8h*_ Meropenem serum concentration 8 h after infusion start, *MIC* Minimum inhibitory concentration

The developed risk assessment tool, the MeroRisk Calculator (beta version), is provided as Additional file 4 and is compatible with Windows operating systems and Excel version 2010 and onwards. When opening the tool, the user might be asked to enable macros, enable content and add to trusted documents. The MeroRisk Calculator is an easy-to-use, three-step Excel spreadsheet (graphical user interface) which can be used to assess the risk of target non-attainment of the PK/PD index 100%T_>MIC_ for non-CRRT patients (Fig. 4a). In step 1, the user provides either the CLCR_CG_ of a patient or its determinants (sex, age, total body weight, serum creatinine concentration), which will then be used to calculate CLCR_CG_. In step 2, the user provides the MIC value of a determined or suspected infecting pathogen, which is used as the target meropenem concentration. In cases in which the MIC value is not available, no MIC value needs to be provided (for handling of blank MIC entry, *see* next step). In step 3, the MeroRisk Calculator computes the probability (“risk”) of target non-attainment for the given CLCR_CG_ and MIC value; if the MIC entry was left blank, the user then has the option to select a EUCAST MIC breakpoint for relevant bacteria [36]. The calculated risk (rounded to integer) of target non-attainment is displayed with the following three-colour coding system: green (≤10%), orange (>10% to < 50%) and red (≥50%). In addition, the tool provides a graphical illustration of the quantified CLCR_CG_-C_8h_ relationship including the 95% prediction interval and predicts, on the basis of provided/calculated CLCR_CG_, the most likely concentration to which meropenem concentrations after multiple dosing will decline before the next dosing (C_8h_) (*see* Fig. 4b; for further details, *see* Additional file 2: Regression model for risk calculation, section 2).

## Discussion

We found a strong relationship between RF and meropenem exposure and consequently PK/PD target attainment, and we developed a graphical user tool to predict the risk of target non-attainment under meropenem standard dosing based on an ICU patient’s RF.

This work was focused on the analysis of the standard dosing regimen for meropenem (1000 mg administered as 30-minute infusions every 8 h) as the approved and still most frequently used dosing regimen in ICUs [12, 45]. To best represent the variety of different ICU patients, the analysis was based on extensively sampled data of a prospective observational study including a large number of patients with highly heterogeneous patient-specific factors from different ICUs, though at one single study centre.

We showed large inter-individual variability in meropenem exposure, which was in accordance with previous studies [22, 23]. The larger variability in concentrations of the late phase compared with the earlier phase of the concentration-time profile (variability: C_min_, C_8h_ > C_4h_) suggested that PK variability was due to variability in drug elimination processes rather than in drug distribution. This finding is supported by population PK analyses that identified larger inter-individual variability on the PK parameter clearance than on volume of distribution [24, 28]. The relatively long observation period of 4 days and the large number of samples collected per patient in our study additionally enabled the quantification of intra-individual variability in meropenem exposure. Its large value led to the hypothesis that meropenem exposure is influenced by certain time-varying patient-specific factors such as confirmed in the present work by longitudinally measured CLCR_CG_.

Our PK/PD analysis demonstrated that meropenem standard dosing did not achieve the desired meropenem PK/PD targets 100%T_>MIC_ and 50%T_>4×MIC_ in a considerable fraction of patients. For pathogens of MIC 2 mg/L, which represents the upper limit of the susceptible range for many important bacteria [36], meropenem exposure was inadequate in every second dosing interval monitored. In line with our work, Carlier et al. found similar results for the target 100%T_>MIC_ given the same MIC value (target attainment 55%) [25]. For infections with less susceptible bacteria of MIC 8 mg/L (I/R breakpoint [36]), which have been shown to commonly occur in ICUs [8, 9], target non-attainment was high, with four of five dosing intervals resulting in sub-therapeutic concentrations (target 100%T_>MIC_). The target attainment analysis with the two targets 100%T_>MIC_ and 50%T_>4×MIC_ revealed similar results. Of note, current knowledge on PK/PD targets for meropenem in heterogeneous ICU populations is limited, and a PK/PD target for this special patient population has not been derived yet. In relation to other PK/PD targets derived for meropenem in diverse clinical studies (e.g., 19.2%T_>MIC_ and 47.9%T_>MIC_ [21], 54%T_>MIC_ [19] and 76-100%T_>MIC_ [20]), the two PK/PD targets selected for our analysis were at the upper end (i.e., stricter). The selection of the higher targets seemed reasonable, given (1) limited knowledge on an adequate PK/PD target for heterogeneous ICU populations and (2) the high severity of illness (median APACHE II_first study day_ 27) and the high proportion of patients with transplants (~58%) in the evaluated population. Indeed, these targets have been reported to be commonly used in clinical practice for ICU patients [40]. However, owing to the limited knowledge of PK/PD targets in ICU patients, there is a crucial need to explore which PK/PD target is best related to clinical outcome in critically ill patients in a prospective clinical trial. Further analyses should also be aimed at investigating differences in PK/PD targets between, for example, different patient sub-groups (e.g., with vs. without transplants), different states of severity of illness or different types of infecting bacteria (gram-positive vs. gram-negative) in a sufficiently large number of patients.

In line with other studies, we identified RF determined by CLCR_CG_ to influence meropenem exposure [26, 27, 29–31]. On the basis of the large number of longitudinally measured meropenem serum concentrations and CLCR_CG_ values covering the full spectrum of RF classes, we were able to quantify a hyperbolic relationship between CLCR_CG_ and meropenem exposure. The present study also included special patient groups such as CRRT and ECMO patients. For CRRT patients, authors of other publications identified measured CLCR determined via 24-h urine collection [28] or residual diuresis [46] as influencing factors on meropenem exposure, both requiring time-consuming urine collection. Although our analysis included a rather small number of CRRT patients, it revealed CLCR_CG_ as a potential determinant of meropenem exposure which can be assessed more easily and quickly in clinical practice than RF markers determined via 24-h urine collection. This finding requires further investigation with a larger number of patients under a well-designed protocol. For the six ECMO patients, the relationship between CLCR_CG_ and meropenem concentrations did not seem different from that of the remaining patients, suggesting that ECMO therapy did not have a strong impact on meropenem serum exposure. This is in line with findings reported by Donadello et al. showing no significant difference between the PK parameters of ECMO and control non-ECMO ICU patients [47].

The impact of RF on the target attainment was overall in accordance with the results of a recent publication by Isla et al. [33], in which the probability of attaining the target 100%T_>MIC_ was analysed for three specific CLCR_CG_ values: Target attainment was 51% for CLCR_CG_ 35 ml/minute (vs. 51% in our study for CLCR_CG_ range 30–59 ml/minute), 3% for CLCR_CG_ 71 ml/minute (vs. 4.6%, 60–89 ml/minute) and 0% for CLCR_CG_ 100 ml/minute (vs. 3.5%, 90–129 ml/minute) for an MIC 8 mg/L. Because the present study included patients covering the full spectrum of RF classes, additional investigation of target attainment in extreme RF classes (severe RI, augmented RF) was possible. For infections with bacteria of MIC 2 mg/L, augmented RF to mild RI was identified as a risk factor of target non-attainment; given bacteria of MIC 8 mg/L, moderate RI was an additional risk factor. These findings imply the need for dosing intensification in patients identified to be at risk of target non-attainment, such as by increasing the dose or prolonged up to continuous infusion, which is currently under clinical investigation; whereas some previous studies have associated continuous infusion with improved clinical cure rates [48, 49], others have not shown a difference in clinical outcome when comparing continuous with intermittent dosing [50]. In this PK/PD analysis, the only patient group that reliably reached the PK/PD targets was the subgroup with severe RI. Notably, these patients also received 1000 mg meropenem every 8 h as 30-minute infusions and thus received higher doses than recommended in the summary of product characteristics (half of indicated dose every 12 h for patients with CLCR_CG_ 10–25 ml/minute [12]).

To enable the practical application of the quantified relationship between RF and meropenem exposure and consequently target attainment, we developed a risk assessment tool in a commonly available and known software (*see* Additional file 4: MeroRisk Calculator, beta version). This easy-to-use Excel tool allows assessment of the risk of target non-attainment for non-CRRT patients displaying RF within a broad range (25–255 ml/minute) and receiving standard dosing of meropenem (1000 mg every 8 h as 30-minute infusions). We implemented the risk of target non-attainment of meropenem depending on creatinine clearance according to the Cockcroft and Gault equation (CLCR_CG_ [34]) and not depending on creatinine clearance determined by 24-h urine collection (CLCR_UC_ [51]), because CLCR_CG_ can be assessed more easily in clinical practice, and the relationship between CLCR_UC_ and meropenem exposure was not better than between CLCR_CG_ and meropenem exposure (*see* Additional file 2: Figure S3). To apply the tool, the user needs to provide only the CLCR_CG_ or its determinants (i.e., sex, age, total body weight and the routinely determined laboratory value serum creatinine). In addition, the MIC value of a bacterium determined or suspected in the investigated patient needs to be provided. Should MIC values not be available, the user has the option to select an MIC breakpoint for important pathogens from the EUCAST database. Because only a limited number of patients with augmented RF or severe RI were included in this analysis, the uncertainty of the CLCR_CG_-meropenem exposure relationship implemented in the MeroRisk Calculator is higher for the extremes of the RF spectrum. Furthermore, the user of the tool needs to keep in mind that in addition to CLCR_CG_, other factors might influence meropenem exposure. To visualise the prediction uncertainty (i.e., uncertainty in the CLCR_CG_-meropenem exposure relationship combined with the variability in C_8h_ values) of the calculated meropenem C_8h_ value for a patients CLCR_CG_, the prediction interval around the CLCR_CG_-meropenem exposure relationship is additionally provided in the risk assessment tool. Of particular note, using the MeroRisk calculator does not require the measurement of a meropenem concentration of a patient. In case of available meropenem concentrations in a patient, use of therapeutic drug monitoring is encouraged to aid therapeutic decision making [52]. The current beta version of the MeroRisk Calculator is intended to be used in the setting of clinical research and training. As a next step, comprehensive prospective validation of the risk calculator in clinical research setting is warranted.

## Conclusions

Our PK/PD analysis demonstrated large inter- as well as intra-patient variability in meropenem serum exposure after standard dosing in critically ill patients. Standard dosing was likely to result in sub-therapeutic meropenem exposure in a considerable fraction of critically ill patients, especially when assuming infections caused by less susceptible bacteria commonly encountered in these patients. CLCR_CG_ was identified as a vital clinical determinant of meropenem exposure and consequently target attainment. In the future, the newly developed risk assessment tool as a graphical user interface (*see* Additional file 4: MeroRisk Calculator) might, if all requirements are met, be beneficial in clinical practice for therapeutic decision making. An ICU patient’s risk of target non-attainment, given his/her RF and the MIC value of the infecting pathogen, would already be accessible when no meropenem concentration measurement is available, such as prior to the start of antibiotic therapy. Our findings indicate that dosing intensification might be needed, depending on a patient’s RF and the susceptibility of the infecting pathogen, and that optimised dosing regimens should be further investigated with respect to increased clinical benefit and reduced development of resistance.

## Additional files

Additional file 1: Study design.pdf. (PDF 170 kb)

Additional file 2: Regression model for risk calculation.pdf. (PDF 475 kb)

Additional file 3: PK/PD target attainment.pdf. (PDF 65 kb)

Additional file 4: MeroRisk Calculator.xltm. (XLTM 330 kb)

## Abbreviations

APACHE II: Acute Physiology and Chronic Health Evaluation II
ARDS: Acute respiratory distress syndrome
BMI: Body mass index
C_4h_: Meropenem serum concentration 4 h after infusion start
C_8h_: Meropenem serum concentration 8 h after infusion start
CLCR_CG_: Creatinine clearance estimated according to Cockcroft and Gault equation
CLCR_UC_: Creatinine clearance determined by 24-h urine collection
C_min_: Minimum meropenem concentration
CRP: C-reactive protein
CRRT: Continuous renal replacement therapy
CV: Coefficient of variation
CVVH: Continuous venovenous haemofiltration
CVVHD: Continuous venovenous haemodialysis
CVVHDF: Continuous venovenous haemodiafiltration
C_X_: Meropenem serum concentrations at specific time points
ECMO: Extracorporeal membrane oxygenation
EUCAST: European Committee on Antimicrobial Susceptibility Testing
I/R: Intermediate/resistant
ICU: Intensive care unit
IL: Interleukin
MIC: Minimum inhibitory concentration
PD: Pharmacodynamic(s)
PK: Pharmacokinetic(s)
RF: Renal function
RI: Renal impairment
S/I: Susceptible/intermediate
SOFA: Sepsis-related Organ Failure Assessment
%T_>MIC_: Percentage of time that drug concentration exceeds the minimum inhibitory concentration
%T_>4×MIC_: Percentage of time that drug concentration exceeds four times the minimum inhibitory concentration

## References

[1] Kempker JA, Martin GS (2016). The changing epidemiology and definitions of sepsis. Clin Chest Med..

[2] Levy Hara G, Kanj S, Pagani L, Abbo L, Endimiani A, Wertheim HFL (2016). Ten key points for the appropriate use of antibiotics in hospitalised patients: a consensus from the Antimicrobial Stewardship and Resistance Working Groups of the International Society of Chemotherapy. Int J Antimicrob Agents..

[3] Kumar A (2010). Early antimicrobial therapy in severe sepsis and septic shock. Curr Infect Dis Rep..

[4] Harbarth S, Garbino J, Pugin J, Romand JA, Lew D, Pittet D (2003). Inappropriate initial antimicrobial therapy and its effect on survival in a clinical trial of immunomodulating therapy for severe sepsis. Am J Med..

[5] MacArthur RD, Miller M, Albertson T, Panacek E, Johnson D, Teoh L (2004). Adequacy of early empiric antibiotic treatment and survival in severe sepsis: experience from the MONARCS trial. Clin Infect Dis..

[6] Roberts JA, Paul SK, Akova M, Bassetti M, De Waele JJ, Dimopoulos G (2014). DALI: defining antibiotic levels in intensive care unit patients: are current β-lactam antibiotic doses sufficient for critically ill patients?. Clin Infect Dis..

[7] Tam VH, Schilling AN, Neshat S, Poole K, Melnick DA, Coyle EA (2005). Optimization of meropenem minimum concentration/MIC ratio to suppress in vitro resistance of *Pseudomonas aeruginosa*. Antimicrob Agents Chemother..

[8] Valenza G, Seifert H, Decker-Burgard S, Laeuffer J, Morrissey I (2012). Mutters R; COMPACT Germany Study Group. Comparative Activity of Carbapenem Testing (COMPACT) study in Germany. Int J Antimicrob Agents.

[9] Cohen J (2013). Confronting the threat of multidrug-resistant Gram-negative bacteria in critically ill patients. J Antimicrob Chemother..

[10] Roberts JA, Abdul-Aziz MH, Lipman J, Mouton JW, Vinks AA, Felton TW (2014). Individualised antibiotic dosing for patients who are critically ill: challenges and potential solutions. Lancet Infect Dis..

[11] De Paepe P, Belpaire FM, Buylaert WA (2002). Pharmacokinetic and pharmacodynamic considerations when treating patients with sepsis and septic shock. Clin Pharmacokinet..

[12] Datapharm. Meronem IV 500 mg & 1 g. Updated 9 Mar 2017. https://www.medicines.org.uk/emc/medicine/11215. Accessed 26 Jun 2017.

[13] Craig WA (1997). The pharmacology of meropenem, a new carbapenem antibiotic. Clin Infect Dis..

[14] Shibayama T, Sugiyama D, Kamiyama E, Tokui T, Hirota T, Ikeda T (2007). Characterization of CS-023 (RO4908463), a novel parenteral carbapenem antibiotic, and meropenem as substrates of human renal transporters. Drug Metab Pharmacokinet..

[15] Christensson BA, Nilsson-Ehle I, Hutchison M, Haworth SJ, Oqvist B, Norrby SR (1992). Pharmacokinetics of meropenem in subjects with various degrees of renal impairment. Antimicrob Agents Chemother..

[16] Roberts DM, Liu X, Roberts JA, Nair P, Cole L, Roberts MS (2015). A multicenter study on the effect of continuous hemodiafiltration intensity on antibiotic pharmacokinetics. Crit Care..

[17] Roehr AC, Frey OR, Koeberer A, Fuchs T, Roberts JA, Brinkmann A (2015). Anti-infective drugs during continuous hemodialysis - using the bench to learn what to do at the bedside. Int J Artif Organs..

[18] Drusano GL (2003). Prevention of resistance: a goal for dose selection for antimicrobial agents. Clin Infect Dis..

[19] Li C, Du X, Kuti JL, Nicolau DP (2007). Clinical pharmacodynamics of meropenem in patients with lower respiratory tract infections. Antimicrob Agents Chemother..

[20] Ariano RE, Nyhlén A, Donnelly JP, Sitar DS, Harding GKM, Zelenitsky SA (2005). Pharmacokinetics and pharmacodynamics of meropenem in febrile neutropenic patients with bacteremia. Ann Pharmacother..

[21] Crandon JL, Luyt C, Aubry A, Chastre J, Nicolau DP (2016). Pharmacodynamics of carbapenems for the treatment of *Pseudomonas aeruginosa* ventilator-associated pneumonia: associations with clinical outcome and recurrence. J Antimicrob Chemother..

[22] Mattioli F, Fucile C, Del Bono V, Marini V, Parisini A, Molin A (2016). Population pharmacokinetics and probability of target attainment of meropenem in critically ill patients. Eur J Clin Pharmacol..

[23] Tsai D, Stewart P, Goud R, Gourley S, Hewagama S, Krishnaswamy S (2016). Optimising meropenem dosing in critically ill Australian Indigenous patients with severe sepsis. Int J Antimicrob Agents..

[24] Jaruratanasirikul S, Thengyai S, Wongpoowarak W, Wattanavijitkul T, Tangkitwanitjaroen K, Sukarnjanaset W (2015). Population pharmacokinetics and Monte Carlo dosing simulations of meropenem during the early phase of severe sepsis and septic shock in critically ill patients in intensive care units. Antimicrob Agents Chemother..

[25] Carlier M, Carrette S, Roberts JA, Stove V, Verstraete A, Hoste E (2013). Meropenem and piperacillin/tazobactam prescribing in critically ill patients: does augmented renal clearance affect pharmacokinetic/pharmacodynamic target attainment when extended infusions are used?. Crit Care..

[26] Kees MG, Minichmayr IK, Moritz S, Beck S, Wicha SG, Kees F (2016). Population pharmacokinetics of meropenem during continuous infusion in surgical ICU patients. J Clin Pharmacol..

[27] Goncalves-Pereira J, Silva NE, Mateus A, Pinho C, Povoa P (2014). Assessment of pharmacokinetic changes of meropenem during therapy in septic critically ill patients. BMC Pharmacol Toxicol..

[28] Isla A (2008). Population pharmacokinetics of meropenem in critically ill patients undergoing continuous renal replacement therapy. Clin Pharmacokinet..

[29] Roberts JA, Kirkpatrick CMJ, Roberts MS, Robertson TA, Dalley AJ, Lipman J (2009). Meropenem dosing in critically ill patients with sepsis and without renal dysfunction: intermittent bolus versus continuous administration? Monte Carlo dosing simulations and subcutaneous tissue distribution. J Antimicrob Chemother..

[30] Alobaid AS, Wallis SC, Jarrett P, Starr T, Stuart J, Lassig-Smith M (2016). Effect of obesity on the population pharmacokinetics of meropenem in critically ill patients. Antimicrob Agents Chemother..

[31] Minichmayr IKM, Roberts JA, Frey OR, Roehr AC, Kloft C, Brinkmann Alexander. Development of a dosing nomogram for continuous infusion meropenem in critically ill patients based on a validated population pharmacokinetic model. J Antimicrob Chemother. 2017 [manuscript submitted for publication]. 10.1093/jac/dkx526 29425283

[32] Crandon JL, Ariano RE, Zelenitsky SA, Nicasio AM, Kuti JL, Nicolau DP (2011). Optimization of meropenem dosage in the critically ill population based on renal function. Intensive Care Med..

[33] Isla A, Canut A, Arribas J, Asín-Prieto E, Rodríguez-Gascón A (2016). Meropenem dosing requirements against Enterobacteriaceae in critically ill patients: influence of renal function, geographical area and presence of extended-spectrum β-lactamases. Eur J Clin Microbiol Infect Dis..

[34] Cockcroft DW, Gault MH (1976). Prediction of creatinine clearance from serum creatinine. Nephron..

[35] Zander J, Maier B, Suhr A, Zoller M, Frey L, Teupser D (2015). Quantification of piperacillin, tazobactam, cefepime, meropenem, ciprofloxacin and linezolid in serum using an isotope dilution UHPLC-MS/MS method with semi-automated sample preparation. Clin Chem Lab Med..

[36] European Committee on Antimicrobial Susceptibility Testing (EUCAST). Breakpoint tables for interpretation of MICs and zone diameters. Version 7.0. 2017. http://www.eucast.org/fileadmin/src/media/PDFs/EUCAST_files/Breakpoint_tables/v_7.1_Breakpoint_Tables.pdf. Accessed 26 Jun 2017.

[37] McKinnon PS, Paladino JA, Schentag JJ (2008). Evaluation of area under the inhibitory curve (AUIC) and time above the minimum inhibitory concentration (T > MIC) as predictors of outcome for cefepime and ceftazidime in serious bacterial infections. Int J Antimicrob Agents..

[38] Taccone FS, Laterre PF, Dugernier T, Spapen H, Delattre I, Wittebole X (2010). Insufficient β-lactam concentrations in the early phase of severe sepsis and septic shock. Crit Care..

[39] Jamal JA, Mat-Nor MB, Mohamad-Nor FS, Udy AA, Wallis SC, Lipman J (2015). Pharmacokinetics of meropenem in critically ill patients receiving continuous venovenous haemofiltration: a randomised controlled trial of continuous infusion versus intermittent bolus administration. Int J Antimicrob Agents..

[40] Wong G, Brinkman A, Benefield RJ, Carlier M, De Waele JJ, El Helali N (2014). An international, multicentre survey of β-lactam antibiotic therapeutic drug monitoring practice in intensive care units. J Antimicrob Chemother..

[41] European Medicines Agency (EMA). Guideline on the use of pharmacokinetics and pharmacodynamics in the development of antibacterial medicinal products. 21 Jul 2016. http://www.ema.europa.eu/docs/en_GB/document_library/Scientific_guideline/2016/07/WC500210982.pdf. Accessed 26 Jun 2017.

[42] European Medicines Agency (EMA). Guideline on the evaluation of the pharmacokinetics of medicinal products in patients with decreased renal function. 20 Feb 2014. http://www.ema.europa.eu/docs/en_GB/document_library/Scientific_guideline/2014/02/WC500162133.pdf. Accessed 26 Jun 2017.

[43] Food and Drug Administration. Guidance for industry: pharmacokinetics in patients with impaired renal function — study design, data analysis, and impact on dosing and labeling. Mar 2010. https://www.fda.gov/downloads/drugs/guidances/ucm204959.pdf. Accessed 26 Jun 2017.

[44] Udy AA, Baptista JP, Lim NL, Joynt GM, Jarrett P, Wockner L (2014). Augmented renal clearance in the ICU. Crit Care Med..

[45] Tabah A, de Waele J, Lipman J, Zahar JR, Cotta MO, Barton G (2015). The ADMIN-ICU survey: a survey on antimicrobial dosing and monitoring in ICUs. J Antimicrob Chemother..

[46] Ulldemolins M, Soy D, Llaurado-Serra M, Vaquer S, Castro P, Rodríguez AH (2015). Meropenem population pharmacokinetics in critically ill patients with septic shock and continuous renal replacement therapy: influence of residual diuresis on dose requirements. Antimicrob Agents Chemother..

[47] Donadello K, Antonucci E, Cristallini S, Roberts JA, Beumier M, Scolletta S (2015). β-Lactam pharmacokinetics during extracorporeal membrane oxygenation therapy: a case-control study. Int J Antimicrob Agents.

[48] Abdul-Aziz MH, Sulaiman H, Mat-Nor MB, Rai V, Wong KK, Hasan MS (2016). B-Lactam Infusion in Severe Sepsis (BLISS): a prospective, two-centre, open-labelled randomised controlled trial of continuous versus intermittent β-lactam infusion in critically ill patients with severe sepsis. Intensive Care Med..

[49] Dulhunty JM, Roberts JA, Davis JS, Webb SAR, Bellomo R, Gomersall C (2013). Continuous infusion of β-lactam antibiotics in severe sepsis: a multicenter double-blind, randomized controlled trial. Clin Infect Dis..

[50] Dulhunty JM, Roberts JA, Davis JS, Webb SAR, Bellomo R, Gomersall C (2015). A multicenter randomized trial of continuous versus intermittent β-lactam infusion in severe sepsis. Am J Respir Crit Care Med..

[51] Levey AS, Inker LA (2017). Assessment of glomerular filtration rate in health and disease: a state of the art review. Clin Pharmacol Ther..

[52] Wicha SG, Kees MG, Solms A, Minichmayr IK, Kratzer A, Kloft C (2015). TDMx: a novel web-based open-access support tool for optimising antimicrobial dosing regimens in clinical routine. Int J Antimicrob Agents..

[53] Knaus WA (1985). APACHE II: a severity of disease classification system Article. Crit Care Med..

[54] Vincent JL, Moreno R, Takala J, Willatts S, De Mendonça A, Bruining H (1996). The SOFA (Sepsis-related Organ Failure Assessment) score to describe organ dysfunction/failure. Intensive Care Med..

