# Supplementary material for: Role of renal function in risk assessment of target non-attainment after standard dosing of meropenem in critically ill patients: a prospective observational study
Source: Crit Care. 2017 Oct 21;21:263. doi: 10.1186/s13054-017-1829-4 (PMC5651591; doi:10.1186/s13054-017-1829-4)
Supplement: Supplementary file 1 — Study design.pdf. (PDF 170 kb) [file 13054_2017_1829_MOESM1_ESM.pdf]

Additional file 1: Study design

The following figure graphically illustrates the study design and the pharmacokinetic sampling schedule described in the main text section 2.1.

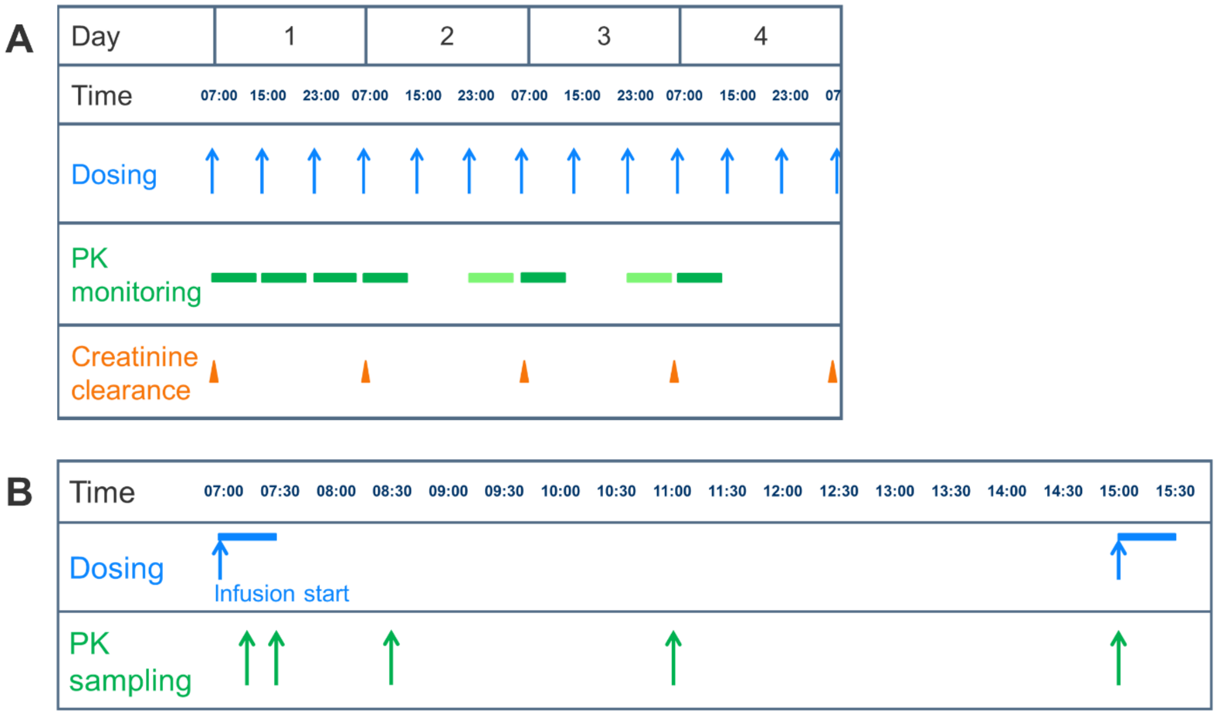

Supplementary figure S1: Study design (A) and sampling schedule of intensively monitored dosing intervals (B). A: *Blue arrows*: Start of meropenem infusions; *Dark green bars*: Intensively monitored dosing intervals; *Light green bars*: Monitored dosing interval with minimum concentration sample only; *Orange triangles*: Determinations of serum creatinine for the estimation of creatinine clearance; **B:** *Blue bars*: Infusion duration (30 min); *Green arrows*: Sampling times (0.25, 0.5, 1.5, 4, 8 h after start of infusion).
