# Supplementary material for: Role of renal function in risk assessment of target non-attainment after standard dosing of meropenem in critically ill patients: a prospective observational study
Source: Crit Care. 2017 Oct 21;21:263. doi: 10.1186/s13054-017-1829-4 (PMC5651591; doi:10.1186/s13054-017-1829-4)
Supplement: Supplementary file 2 — Regression model for risk calculation.pdf. (PDF 475 kb) [file 13054_2017_1829_MOESM2_ESM.pdf]

Additional file 2: Regression model for risk calculation

1. Regression model

The relation between $CLCR_{CG}$ and the observed $C_{8h,\,obs}$ meropenem values allowing to predict a typical $C_{8h,\,pred}$ value ($C_{8h,\,pred} = \alpha \cdot \frac{1}{(CLCR_{CG})^{\beta}}$, for derivation see main text section 2.3) was quantified in a weighted linear model on double natural logarithmic scale, i.e.

$$\ln(C_{8h,\,obs}) = a + b \cdot \ln(CLCR_{CG}) + \varepsilon,$$

in which $a = \ln(\alpha)$, $b = -\beta$. The residual variability ε represents the difference between the logarithmised observed $C_{8h,\,obs}$ values and the logarithmised model-predicted typical $C_{8h,\,pred}$ values and is assumed to be normally distributed with variance $\hat{\sigma}^2_{\varepsilon}$ proportional to $CLCR_{CG}$.

2. Confidence and prediction intervals of C_{8h} values and risk of target non-attainment

We denoted the model-predicted typical value by $\ln(C_{8h,\,pred}) = \hat{a} + \hat{b} \cdot \ln(CLCR_{CG})$ ($\hat{a}$, $\hat{b}$ are estimated regression model parameters). Confidence intervals were used to indicate the uncertainty in this quantified relationship. These were derived from classic theory of linear models [1], based on a regression variability parameter $\hat{\sigma}^2_{reg}$ determined from $\hat{a}$, $\hat{b}$ and due to heteroscedasticity varying with the value of $CLCR_{CG}$.

In addition, to determine the range of plausible C_{8h} values for a patient cohort with a specific $CLCR_{CG}$, prediction intervals were constructed, again using classic theory of linear models [1]. The prediction variability $\hat{\sigma}^2$ around the typical $C_{8h,\,pred}$ value consisted of the sum of two components: the regression variability $\hat{\sigma}^2_{reg}$ and the residual variability $\hat{\sigma}^2_{\varepsilon}$.

To obtain - from the prediction variability $\hat{\sigma}^2$ - prediction intervals and the risk of target non-attainment, standardised residuals were utilised (again part of the classic theory of linear models [1]). The standardised residuals $\frac{\ln(C_{8h,\,obs}) - \ln(C_{8h,\,pred})}{\hat{\sigma}}$ are t-distributed with n-2 degrees of freedom, with n being the number of data points used in the regression analysis.

The 95% prediction interval (PI) and risk of target non-attainment $P(C_{8h} \leq MIC)$ were then derived from quantiles $q_{\alpha}^{t_{n-2}}$ and the cumulative distribution function $F^{t_{n-2}}$ of the t-distribution, i.e.

$$95\%\ PI=\left[C_{8h,\ pred} \cdot \exp\left(\hat{\sigma} \cdot q_{0.025}^{t_{n-2}}\right);\ C_{8h,\ pred} \cdot \exp\left(\hat{\sigma} \cdot q_{0.975}^{t_{n-2}}\right)\right]$$

and

$$P(C_{8h} \leq MIC)=F^{t_{n-2}}\left(\frac{\ln(MIC) - \ln(C_{8h,\ pred})}{\hat{\sigma}}\right).$$

Computations were carried out in R, version 3.3.2 (R Foundation for Statistical Computing, Vienna, Austria) using functions for linear models.

3. Goodness of fit of the regression model

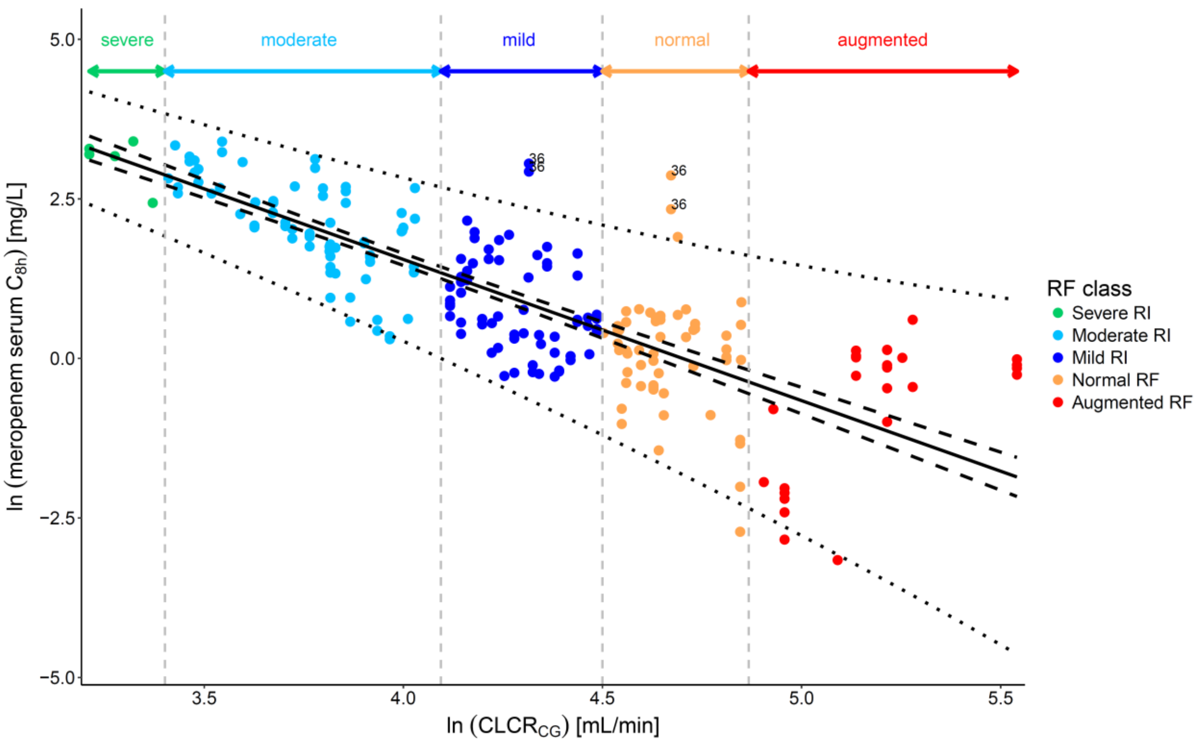

Supplementary Figure S1: Relation between meropenem serum concentrations and creatinine clearance. Logarithmised meropenem serum concentrations 8 h after start of infusion (C_{8h}) in non-CRRT patients versus logarithmised $CLCR_{CG}$ are shown. *Colour of symbols*: Respective renal function (RF) class of a patient at time of determined C_{8h} value; *Dashed vertical lines/horizontal arrows*: Separation of renal function classes; *Data points labelled with 36*: Four C_{8h} values of patient 36; *Black solid line:* Quantified ln($CLCR_{CG}$) - ln(C_{8h}) relationship (representing ln($C_{8h,\ pred}$), excluding data of patient 36); *Black dashed/dotted lines:* 95% confidence interval/95% prediction interval (excluding data of patient 36).

Abbreviations: *$CLCR_{CG}$*: Creatinine clearance estimated according to Cockcroft and Gault [2]; *CRRT*: Continuous renal replacement therapy; *C_{8h}*: Meropenem serum concentration 8 h after start of infusion; RF: renal function; RI: Renal impairment.

4. Comparison of other scenarios

4.1. Model predictions for meropenem concentrations including or excluding patient 36 in model parameter estimation

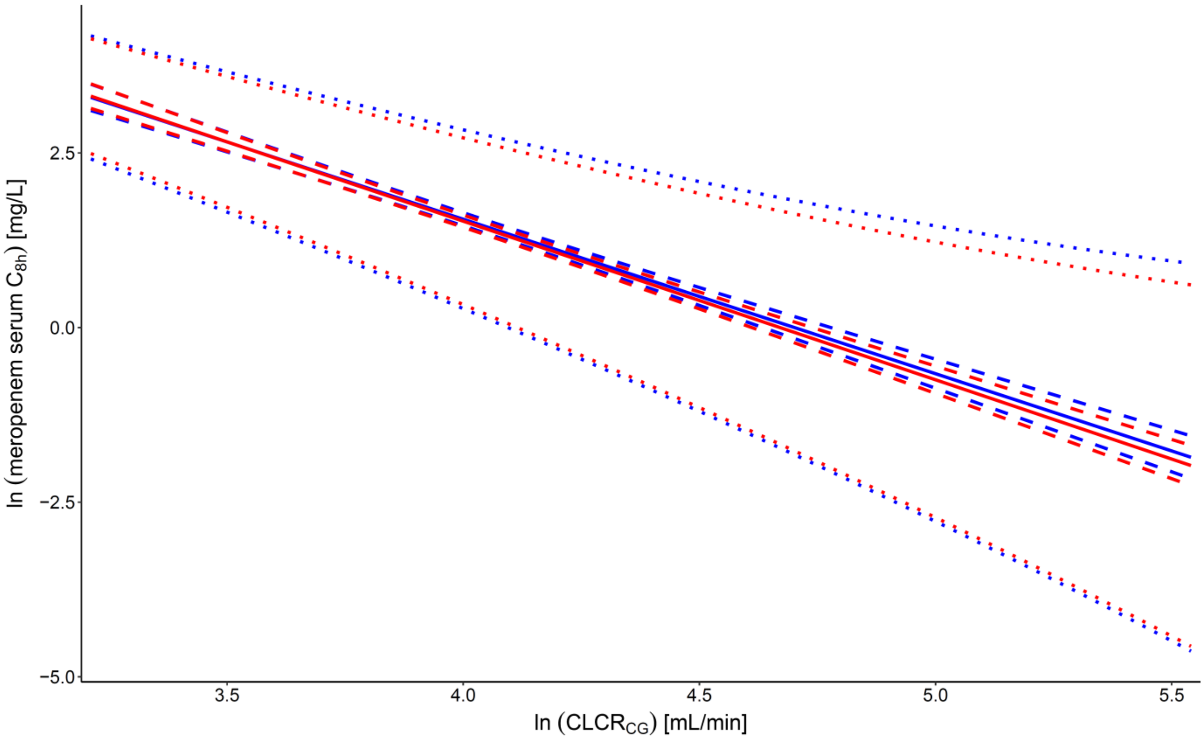

Supplementary Figure S2: Comparison of C_{8h} meropenem predictions including or excluding patient 36 in model parameter estimation. *Solid/dashed/dotted lines:* Quantified ln($CLCR_{CG}$) - ln(C_{8h}) relationship/95% confidence interval/95% prediction interval; *Red:* Excluding patient 36; *Blue:* Including patient 36.

Abbreviations: *$CLCR_{CG}$*: Creatinine clearance estimated according to Cockcroft-Gault [2]; *C_{8h}*: Meropenem serum concentration 8 h after start of infusion.

4.2. Comparing the regression model using creatinine clearance according to Cockcroft and Gault ($CLCR_{CG}$) with the regression model using creatinine clearance determined via 24 h urine collection ($CLCR_{UC}$)

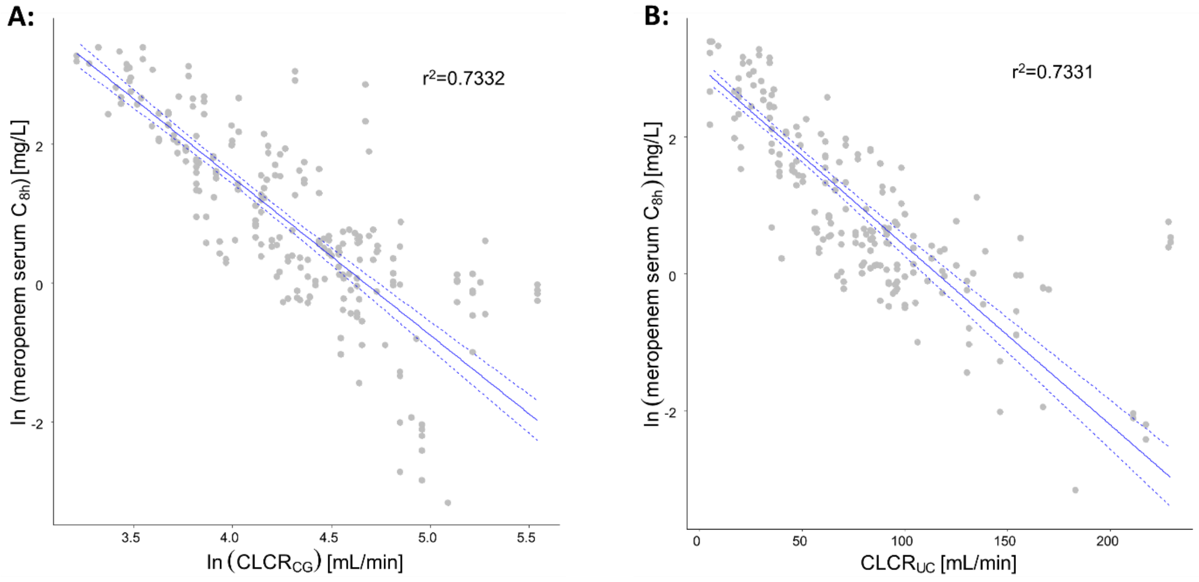

Supplementary Figure S3: Relation between meropenem serum C_{8h} and $CLCR_{CG}$ (A), and $CLCR_{UC}$ (B). *Blue solid line*: Quantified relationship between renal function marker and meropenem serum C_{8h}. The relationship was quantified using a weighted (1/CLCR) linear least square regression on (A) double logarithmic ($\ln(CLCR_{CG})$ and $\ln(C_{8h})$) and (B) semi-logarithmic scale ($CLCR_{UC}$ and $\ln(C_{8h})$) for $CLCR_{CG}$ and $CLCR_{UC}$, respectively; *Blue dotted line*: 95% confidence interval around relationship.
Abbreviations: $CLCR_{CG}$: Creatinine clearance estimated according to Cockcroft and Gault [2]; $CLCR_{UC}$: Creatinine clearance determined using 24-hour urine collection [3]; C_{8h}: Concentration at 8 h after infusion start.

References

[1] Montgomery DC, Peck EA, Vining GG. Introduction to Linear Regression Analysis. 5th ed. New York: Wiley; 2012.

[2] Cockcroft DW, Gault MH. Prediction of Creatinine Clearance from Serum Creatinine. Nephron. 1976;16:31–41.

[3] Levey AS, Inker LA. Assessment of Glomerular Filtration Rate in Health and Disease: A State of the Art Review State of the Art Review for Clinical Pharmacology and Therapeutics. Clin. Pharmacol. Ther. 2017;102:405–19.
