# Supplementary material for: Role of renal function in risk assessment of target non-attainment after standard dosing of meropenem in critically ill patients: a prospective observational study
Source: Crit Care. 2017 Oct 21;21:263. doi: 10.1186/s13054-017-1829-4 (PMC5651591; doi:10.1186/s13054-017-1829-4)
Supplement: Supplementary file 3 — PK/PD target attainment.pdf. (PDF 65 kb) [file 13054_2017_1829_MOESM3_ESM.pdf]

Additional file 3: PK/PD target attainment

The following tables summarise the attainment of the pharmacokinetic/pharmacodynamic (PK/PD) targets 50%$T_{>4xMIC}$ and 100%$T_{>MIC}$ evaluated for all patients not undergoing continuous renal replacement therapy (non-CRRT, n=42) on sample level (n=233) for the MIC range from 0.25 mg/L to 8 mg/L for the dosing regimens:

- 1000 mg meropenem administered as 30-min i.v. infusion every 8 hours (s. Supplementary Table 1)
- 2000 mg meropenem administered as 30-min i.v. infusion every 8 hours (s. Supplementary Table 2)

Supplementary Table S1: PK/PD target attainment for all non-CRRT patients after standard meropenem dosing (1000 mg, i.v. 30 min, every 8 h) for different MIC values.

MIC [mg/L]	PK/PD target attainment, %	
	50%$T_{>4xMIC}$	100%$T_{>MIC}$
	C_{4h} ≥ 4xMIC, %	*C_{4h} ≥ MIC, %*
0.25	99.6	95.5
0.5	96.9	91.9
1	91.0	78.0
2	56.1	48.4
4	27.4	38.1
8	7.17	20.6

Abbreviations: C_X: Meropenem serum concentration at specific time point X of concentration-time profile; MIC: Minimum inhibitory concentration; PK/PD: Pharmacokinetic/pharmacodynamic.

Colour coding: Target attainment >90% (green), 50-90% (orange), <50% (red).

Supplementary Table S2: PK/PD target attainment for all non-CRRT patients extrapolated to intensified meropenem dosing (2000 mg, i.v. 30 min, every 8 h) for different MIC values.

MIC [mg/L]	PK/PD target attainment, %	
	50%$T_{>4xMIC}$	100%$T_{>MIC}$
0.25	100	97.3
0.5	99.6	95.5
1	96.9	91.9
2	91.0	78.0
4	56.1	48.4
8	27.4	38.1

Abbreviations, Colour coding: s. Supplementary table 1.
